# Editorial: Professional identities within healthcare professions education

**DOI:** 10.3389/fmed.2025.1585580

**Published:** 2025-04-17

**Authors:** Adam P. Sawatsky, Lynn V. Monrouxe

**Affiliations:** ^1^Mayo Clinic, Rochester, MN, United States; ^2^Faculty of Medicine and Health, School of Health Sciences, The University of Sydney, Camperdown, NSW, Australia

**Keywords:** professional identity formation (PIF), healthcare profession education, belonging, professional identities, wellbeing

Welcome to the Research Topic, *Professional Identities Within Healthcare Professions Education*. In this Research Topic, we include 6 articles exploring different aspects of professional identity formation in healthcare professions education. The included articles cover a wide range of Research Topics, including the conceptualization of professional identity formation (Sternszus et al.) the construction of female surgeons' intersecting identities (Offiah et al.) the measurement of professional identities (Ryan et al., Zeng et al., Chang et al.) and the report of a program to transform the professional identities of medical teachers (Kiran et al.).

Underlying all scholarly work on professional identity formation lies some foundational assumptions, either implicit or explicit, about the nature of *identities* (1). Theories of identity seek to answer the fundamental question “Who am I?” and address both personal and social realities of the answers to that question: namely one's beliefs about ourselves and the relationships and social groups we identify with (2). The role of identities takes on additional meaning when added to the word *professional*. Being a professional implies a certain level of trust, undergirded by a commitment to the quality of and moral involvement in one's work (3). Therefore, linked to the idea of a profession is the notion of identity—the shared values and norms of individuals who belong to the profession and which guide their work (3, 4). This then leads to the notion of *formation*. Theories describe how identities are discovered, constructed, and change over time—all processes that researchers in the healthcare professions have named *professional identity formation*.

While this notion of professional identity formation has been effectively woven into the fabric of medical education (5), researchers continue to attempt to untangle the complex concepts woven into the body of research on the Research Topic (6). The articles in this Research Topic nicely illuminate several important issues that become apparent when viewed through this lens. First, identities provide a lens into the interaction between individuals and context, particularly within the training environment. Sternszus et al. highlight how the socialization process inherent in professional identity formation cannot be one of social reproduction, but needs to entail a critical examination of the underlying assumptions laden within the social construct of a healthcare professional and the context that shapes that identity formation. Offiah et al. further this point as they examine the harmful stereotypes associated with gender and race—exploring the intersectionality of these identities provides a window for thinking about how we might provide more flexible training and support to allow for a sense of belonging to the profession.

Second, identities and their formation can inform the discussion of professional struggle and burnout (7, 8). In addition to the discussion about context and intersectionality above (Sternszus et al. and Offiah et al.), these studies link professional identities to the concepts of psychological distress, burnout, and resilience (Ryan et al., Zeng et al.). Indeed, a stronger sense of professional identity is associated with measures of growth (Chang et al.) and decrease in burnout (Zeng et al.). While these studies are cross-sectional, they point to links between the concepts of professional identities and an improved sense of wellbeing within one's work.

Third, these studies highlight the importance of community and support in the development of a professional identity. Kiran et al. demonstrate that developing an identity is a transformative journey—one that requires a supportive environment to challenge one's underlying assumptions about self and the profession. This highlights that if the goal of healthcare professions education is identity formation, then time, resources, and support are required to properly educate professionals.

While these articles highlight important points about professional identity formation in healthcare professions education, we raise several questions for educators and researchers to consider. First, what is meant by a *strong* professional identity? We believe that this idea links to a psychological understanding of an achieved identity—one that is arrived at through adequate exploration and strong commitment to that identity (9). While this is a dominant way of conceptualizing identities in the healthcare professions, we want to recognize that identities are often being challenged and reimagined throughout one's lifetime. We urge care in labeling an identity as strong, being careful to examine the assumptions that label carries. Second, what are the implications for trying to *measure* professional identities? This is partially related to the first question—how are we measuring identity and what does that mean about the strength or weakness of identity? It also raises implications about how these measures will be used in clinical and educational practices. While the articles in this group provided some arguments for the validity of measures of professional identity, we would urge for more research to examine how these measurements change over time, and consider the implications of using these measures within educational settings. Last, these articles challenge our understanding of the relationship between identity and belonging. While many of these articles call for greater inclusion in the healthcare professions, educators need to grapple with the question: how far can we change an environment to be more inclusive until we lose the distinctives of the profession? Additionally, who decides where that line is drawn? This is a complex negotiation between all of us, including society and patients. We hope that these questions will guide future work on professional identity formation within healthcare professions education.

Taken together, this group of articles provide us with a wonderful exploration into the concept of professional identities and how they are discovered, developed, and performed in the context of healthcare professions education. The insights and questions derived from these articles will continue to advance the discussion of professional identity formation.
